# Influence of *Lactobacillus casei* WB 315 and crude fish oil (CFO) on growth performance, EPA, DHA, HDL, LDL, cholesterol of meat broiler chickens

**Published:** 2020-04

**Authors:** Andreas Berny Yulianto, Widya Paramita Lokapirnasari, Rifqi Najwan, Hana Cipka Pramuda Wardhani, Nabil Fariz Noor Rahman, Khoirul Huda, Nuria Ulfah

**Affiliations:** 1 Department of Animal Husbandry, Faculty of Veterinary Medicine, Universitas Airlangga, Surabaya, Indonesia; 2 Department of Animal Husbandry, Faculty of Veterinary Medicine, Universitas Airlangga, Surabaya, Indonesia; 3 Department of Animal Husbandry, Faculty of Veterinary Medicine, Universitas Airlangga, Surabaya, Indonesia

**Keywords:** *Lactobacillus casei*, Crude fish oil, Eicosapentaenoic acid, Docosahexaenoic acid, Broiler performance

## Abstract

**Background and Objectives:**

Use of antibiotics as growth promoters in animal feeds has been restricted due to the residues in poultry products such as egg and meat, furthermore to the antibiotic resistant of pathogenic bacteria. The prohibition of their use opens the opportunity for the use of non-antibiotic feed additives such as probiotics. The objectives of this study were to investigate the effect of the addition of *Lactobacillus casei* WB 315 and crude fish oil (CFO) to diets on growth performance, eicosapentaenoic acid (EPA), docosahexaenoic acid (DHA), low density lipoproteins (LDL), high density lipoprotein (HDL), and cholesterol levesl of broiler chickens.

**Materials and Methods:**

In this research, one-day old male broiler chicks were used and divided equally into four groups, namely a basal diet without *L. casei* WB 315 and without CFO (P0), basal diet supplemented with 0.5% *L. casei* WB 315 of total broiler basal feed (1.2 × 10^9^ cfu/ml) and without CFO (P1), basal diet supplemented without *L. casei* WB 315 and 1% CFO of total broiler basal feed (P2), and basal diet supplemented with 0.5% *L. casei* WB 315 of total broiler basal feed (1.2 × 10^9^ cfu/ml) and 1% CFO of total broiler basal feed (P3) for 35 days.

**Results:**

The results of addition 0.5% *Lactobacillus casei* WB 315 (1.2 × 10^9^ cfu/ml) and 1% CFO of total broiler basal feed after 35 days showed significant difference among treatment in feed efficiency (p<0.05), feed conversion ratio (p<0.05), feed consumption (p<0.05), EPA (p<0.05), DHA (p<0.05), increase HDL (p<0.05), reduced the LDL (p<0.05), and reduce cholesterol (p<0.05) in meat broiler chicken.

**Conclusion:**

It is concluded that the addition of *L. casei* WB 315 and crude fish oil (CFO) could significant improve the growth performance (feed efficiency, feed conversion ratio, feed consumption) and could significantly improve EPA, DHA and increase HDL and decrease LDL in meat poultry product.

## INTRODUCTION

Probiotics are live microbes which when administrated in adequate quantities can promote and improve the host health and growth (1–6). Probiotics have specific properties such as resistance to bile salts and acid, stable viability, ability to adhere in mucosa of digestive tract. The most frequently used probiotics in poultry rations are *Lactobacillus*. The broilers fed with *Lactobacillus* showed significant increased of body weight (p<0.05) in both the grower and the finisher periods. Jin et al. (1997) and Mohan et al. (1996) reported that positive effects of probiotics in broiler chickens occurred only after the fourth week of growth at 0–6 weeks old chicks (7, 8). The objective of the study was to investigate the effect of *L. casei* WB 315 and Crude Fish Oil (CFO) to diets on feed intake (FI), feed conversion ratio (FCR), feed efficiency (FE), EPA, DHA, HDL, LDL and cholesterol of broilers.

## MATERIALS AND METHODS

### Bacterial strain.

The *Lactobacillus casei* WB 315 used in this study was isolated, identified and assessed the probiotic properties *in vitro* and showed that *L. casei* WB 315 was resistant to low pH by testing its survivability with acid and grow under conditions simulating the intestinal environment (by testing its survivability with bile salt) and to inhibit the growth of *Eschericia coli* and *Staphylococcus aureus* as shown by antibacterial activity against *E. coli* and *S. aureus*). *L. casei* WB 315 was added to the basal diet containing 1.2 × 10^9^ cfu/ml per day for 35 days. Dosage of *L. casei*: 0.5% of total broiler feed. Dosage of crude fish oil (CFO): 1% of total broiler feed.

### In vivo study.

There were four groups of treatment, control (P0), a basal diet without *L. casei* WB 315 and CFO, P1: basal diet supplemented with 0.5% *L. casei* WB 315 of total broiler feed and without CFO, P2: basal diet supplemented without *L. casei* WB 315 and 1% CFO, and P3: basal diet supplemented with 0.5% *L. casei* WB 315 of total broiler feed and 1% CFO for 35 days. The chicks were housed in individual cage, feed and water were provided ad libitum. All diets were antibiotic-free and formulated to meet the nutrient requirements for broilers. The growth performance parameters were recorded weekly, include: feed intake (FI), feed conversion ratio (FCR), feed efficiency (FE).

### Statistical analysis.

All data were analysed using the Analysis of Variant (ANOVA) procedure in a completely randomized design. Furthermore, the differences among all treatments were continued by Duncan’s multiple range tests. Results expressed as p<0.05 were considered significant.

## RESULTS

### Effect of CFO and *L. casei* WB 315 on feed intake, feed conversion ratio, feed efficiency.

The results of feed intake showed that there were significant differences between treatments (p<0.05) compared to control. The P0 treatment was significantly different (p<0.05) with P1, P2 and P3. P1 treatment showed significant differences (p<0.05) with treatment P0 and P2, P3. P2 treatment was not significantly different (p> 0.05) with P3 treatment, but P2 and P3 treatments were significantly different (p<0.05) with treatments P1 and P0. The highest feed intake results were found in treatment P0 (86.51 g/chick/day), while the lowest feed intake was found in P2 and P3 treatments, i.e 79.50 and 79.31 g/chick/day, respectively. The data of feed intake are listed in Table 1.

**Table 1.** The effect of *L. casei* WB 315 and CFO on feed intake, feed conversion ratio, fed efficiency

**Treatment**	**Feed intake (g/chick/day) ± SD**	**Feed conversion ratio ± SD**	**Feed efficiency (%) ± SD**
P0: 0% *L. casei* + 0% CFO	86.51^c^ ± 0.78	2.12^b^ ± 0.06	47.06^a^ ± 1.18
P1: 0.5% *L. casei* + 0% CFO	81.24^b^ ± 0.64	1.99^a^ ± 0.05	50.27^b^ ± 1.29
P2: 0% *L. casei* + 1% CFO	79.50^a^ ± 1.49	1.94^a^ ± 0.13	51.64^b^ ± 3.46
P3: 0.5% *L. casei* +1% CFO	79.31^a^ ± 1.01	1.98^a^ ± 0.06	50.54^b^ ± 1.45

(a, b, c) Means in the same column with the different superscript are significantly different (p<0.05).

The results of the feed conversion ratio in broilers showed that there were significant differences (p<0.05) between treatment and control. The P0 treatment was significantly different (p<0.05) with treatments P1, P2 and P3, whereas between treatments P1, P2 and P3 did not show significant differences (p> 0.05). The highest feed conversion ratio results in treatment P0 (2.12), while low feed conversion is found in treatments P1, P2 and P3. A low FCR value illustrates that feed efficiency is high because lower feed consumption results in higher meat production (Table 1).

The results of the feed efficiency on broilers showed that there were significant differences (p<0.05) between treatment and control. The P0 treatment was significantly different (p<0.05) with treatments P1, P2 and P3, whereas between treatments P1, P2 and P3 did not show significant differences (p>0.05). The lowest feed efficiency results in treatment P0 (47.06%), while high feed efficiency is found in treatments P1, P2 and P3. High feed efficiency values illustrate that with lower feed consumption, but produce higher meat production. Data from the measurement of feed efficiency on treatment are listed in Table 1.

### The influence of CFO and *L. casei* WB 315 on EPA, DHA in broilers meat.

The results of the study of EPA content in meat broiler showed significant differences (p<0.05) between treatments with control. The P0 treatment showed significant differences (p<0.05) with treatments P1, P2 and P3. The EPA content in treatments P3 and P1 did not show significant differences (p>0.05), but P3 and P1 showed differences (p<0.05) with P0 and P2. The EPA content in P2 treatment showed significant differences (p<0.05) with treatments P0, P3 and P1. The lowest EPA content was found in the control treatment (P0), while the highest EPA content was found in the treatment of 1% CFO (P2). The data of EPA was shown in Table 2.

The results of assesment the DHA content in treated chicken showed a difference (p<0.05) between treatment and control. P0 treatment was not different (p>0.05) with treatment P3 and P1, but treatment P0 was significantly different (p<0.05) with the treatment of 1% CFO (P2). The highest DHA content was found in the treatment of 1% CFO (P2) which was 2.57%, while the low DHA content was found in treatments P1, P3 and P0, which was 1.22%, 0.75% and 0.31% respectively. Data from the measurement of DHA on treatment are listed in Table 2.

**Table 2.** The effect of CFO and *L. casei* WB 315 on EPA, DHA

**Treatment**	**EPA (%) ± SD**	**DHA (%) ± SD**
P0: 0% *L. casei* WB 315 + 0% CFO	0.19^a^ ± 0.08	0.31^a^ ± 0.011
P3: 0.5% *L. casei* WB 315 + 1% CFO	0.68^b^ ± 0.00	0.75^a^ ± 0.33
P1: 0.5% *L. casei* WB 315 + 0% CFO	0.77^b^ ± 0.27	1.22^a^ ± 0.39
P2: 0% *L. casei* WB 315 + 1% CFO	2.33^c^ ± 0.00	2.57^b^ ± 1.48

(a, b, c) Means in the same column with the different superscript are significantly different at (p<0.05).

### The influence of CFO and *L. casei* WB 315 on HDL, LDL, cholesterol.

The results of HDL content in the treatment showed that there were significant differences (p<0.05) between treatment and control. The HDL content in the control treatment (P0) showed significant differences (p<0.05) with treatments P1, P2 and P3. The HDL content in treatment P1 showed significant differences (p<0.05) with treatments P0, P2 and P3. The HDL content in P2 treatment showed significant differences (p<0.05) with P0, P1 and P3, ie 1% CFO of total basal diet showed HDL content more higher compared to treatments P1 and P0. The HDL content in P2 treatment showed significant differences (p<0.05) with treatment P3, P1 and P0. The P3 treatment ie 0.5% *L. casei* WB 315 from basal total feed and 1% CFO from basal total feed showed more higher HDL (28.05%) compared to treatment P2, P1 and P0. The lowest HDL content was found in the control treatment (8.68%), while the highest HDL content was found in the P3 treatment (28.05%) (Table 3).

**Table 3.** The effect of CFO and *L. casei* WB 315 on HDL, LDL, Cholesterol

**Treatment**	**HDL (%) ± SD**	**LDL (%) ± SD**	**Cholesterol (mg/dL) ± SD**
P0: 0% *L. casei* WB 315 + 0% CFO	8.68^a^ ± 0.80	88.19^d^ ± 0.78	112.25^d^ ± 1.26
P1: 0.5% *L. casei* WB 315 + 0% CFO	13.32^b^ ± 0.74	83.90^c^ ± 0.83	108.60^c^ ± 1.00
P2: 0% *L. casei* WB 315 + 1% CFO	22.38^c^ ± 0.78	64.24^b^ ± 0.78	104.53^b^ ± 0.96
P3: 0.5% *L. casei* WB 315 + 1% CFO	28.05^d^ ± 0.82	57.51^a^ ± 0.82	86.87^a^ ± 0.78

(a, b, c) Means in the same column with the different superscript are significantly different at (p<0.05).

The results of assay the LDL content in the treatment showed a significant difference (p<0.05) between treatment and control. The LDL content in the control treatment (P0) showed significant differences (p<0.05) with treatments P1, P2 and P3. The LDL content in treatment P1 showed significant differences (p <0.05) with treatments P0, P2 and P3. The LDL content in P2 treatment showed a significant difference (p<0.05) with P0, P1 and P3, ie the treatment of 1% CFO from the total basal diet showed more lower LDL content compared to treatments P1 and P0. The LDL content in P2 treatment showed significant differences (p<0.05) with treatment P3, P1 and P0. The LDL content in treatment P3 showed significant differences (p<0.05) with treatment P2, P1 and P0, ie treatment of 0.5% *L. casei* WB 315 from total basal feed and 1% CFO from total basal feed described more lower LDL content compared with treatment P2, P1 and P0. The highest LDL content was found in the control treatment (88.19%), while the lowest LDL content was found in the P3 treatment (57.51%) (Table 3).

The results of cholesterol content in the treatment showed a significant difference (p<0.05) between treatment and control. The cholesterol content in the control treatment (P0) showed significant differences (p<0.05) with treatments P1, P2 and P3. The cholesterol content in treatment P0 is more higher than the treatment P1, P2 and P3. The cholesterol content in treatment P1 shows that there are significant differences (p<0.05) with treatments P0, P2 and P3. The cholesterol content in treatment P1 shows more lower than control. The cholesterol content in P2 treatment showed significant differences (p<0.05) with P0, P1 and P3. The cholesterol content of treatment P2 shows more lower than P1 and P0. The cholesterol content in treatment P3 shows that there is a significant difference (p<0.05) with treatment P2, P1 and P0, namely treatment of 0.5% *L. casei* WB 315 from basal total feed and 1% CFO from basal total feed describe more lower cholesterol content than treatment P2, P1 and P0. The highest cholesterol content was found in the control treatment (112.25%), while the lowest cholesterol content was found in the P3 treatment (86.87%) (Table 3).

## DISCUSSION

### Effect of CFO and *L. casei* WB 315 on levels of feed intake, feed conversion ratio, feed efficiency.

*L. casei* WB 315 is more economically valuable than that of the control due to the lower consumption levels, but it is capable of producing relatively the same weight gain so as to produce a better feed conversion ratio. Feed consumption is important, because it refers to the fulfillment of the need for both basic living and production. Good feed consumption will give the body a chance to retain its nutrients and the useful proteins derived from food substances more, so that the body’s needs for protein are fulfilled (9).

The result of this present study agreement with Lopez (2001), that supplementation fish oil could increase the weight gain and improve feed conversion ratios of broiler than the control diet and did not cause adverse effects on mortality (10). Crittenden et al. (2005) determined that probiotic have beneficial impacts on the commercial animals by enhancing body weight gain and improving feed conversion and feed efficiency (11). Mechanism of probiotic *Lactobacillus* sp. to improve feed intake and feed efficiency through influence of the crypt depth and villi height in the small intestine of broilers chicken (12). Samaya and Yamauchi (2002) stated that the administration of *Lactobacillus sakei* Probio-65 increased villi height and crypt depth in jejunum of broilers as compared to chickens fed with antibiotic and chickens that were fed with feed without of antibiotics or probiotics. Probiotics could increase the length of villi by activating cell mitosis and induce gut epithelial-cell proliferation (13). Increased of villi height is beneficial to the broilers as the increased surface area of the villi enhanced the absorption of nutrient (14). Deeper crypt depth by probiotics allow higher turnover rate of villi tissue and replenish villi which may lost due to sloughing or inflammation in response to pathogen infection (15). The enhanced absorption of nutrient in the intestinal epithelium may lead to digestive enzymes secretion in the GI tract and eventually increase growth broilers performance.

The results of the analysis of variance demonstrated that the use of CFO and *L. casei* WB 315 on ration resulted in significant differences among treatments (p<0.05) on feed conversion ratio (FCR). The highest FCR was obtained using P0 (2.12), while the lower ones were from P2 (1.94), P3 (1.98) and P1 (1.99). The doses of 0.5% *L. casei* WB 315 and additional CFO in this study produced feed conversion rates that were 8.49% lower than that of the control. These results were in line with the research by Carragher who stated that the feed containing omega-3 had a significant (10%) lower feed conversion ratio and a mortality rate that was not different from that of the control diet (16), he also stated that the increase in body weight of the broilers that were fed diets supplemented with *Lactobacillus* was consistent in both the grower and the finisher periods (17). The good effects of probiotics in chickens occurred only later on the fourth week of growth (18). This effect was the increased intestinal amylase enzyme activity and growth of the Lactobacilli colonizing effect in the intestine (19).

Based on the research that has been conducted, an average feed efficiency (FE) was obtained for each treatment presented in Table 1. The results of analysis of variance showed that the use of *L. casei* WB 315 on ration treatments led to major differences among treatments (p<0.05) on the feed efficiency as they demonstrated that the highest feed efficiency was in P1 (50.27%), P2 (51.64%) and P3 (50.54%), while the lowest was discovered in P0 (47.06%). Probiotics can be utilized to manipulate the ecosystem in the digestive tract of cattle in order for their absorption process to work well and suffice to suppress pathogenic bacteria. They can also be used to minimize the feed consumption by increasing the population of beneficial microbes for livestock and preventing the growth of harmful microbes in the digestive tract, so that it can improve the digestion of feed (9). The performance results of the experiment revealed that the dietary supplementation with *Lactobacillus* enhances broiler performance (20, 21) and this result is similar to the findings of (18), who reported that the good effect of probiotics in chickens occurred only later on the fourth week of growth, but it is in contrast with the finding of (22), who noted that the average daily gain of broilers fed probiotics was significantly increased during the starter period, but not during the finisher period. The present study agreement with the other research that supplementation *Lactobacillus* to broilers fed also resulted in higher broiler daily weight gain, feed efficiency, and reduce mortality (23), improved feed efficiency, feed intake and carcass yield of broilers (24).

### The influence of CFO and *L. casei* WB 315 on levels of EPA, DHA.

The results of the analysis of variance indicated that the use of CFO and *L. casei* WB 315 on ration treatments resulted in significant differences among treatments (p<0.05) on levels of DHA in chicken meat. They also indicated that the highest level of DHA was present in P2 (2.57%), while the lowest were produced in P1 (1.22%), P3 (0.75%) and P0 (0.31%). These results are related to the other research, which states that feed containing omega 3 can significantly increase the content of omega 3, EPA and DHA in meat and eggs, this is because most of EPA and DHA were being deposited in the phospholipid fraction (16). The fatty acid composition of broiler meat may be modified by changing the fatty acid composition of the chicken feed formulation (25, 26).

The result of this present study agreement with several previous study. In poultry, the consumption of diets high in PUFA, particularly eicosapentaenoic acid (EPA) and docosahexaenoic acid (DHA), it has been demonstrated to improve body weight gain (27, 28). The enrichment n-3 PUFA containing fish oil at 2% and 4% in broiler diets showed increased accumulation of n-3 LC-PUFA in muscle and adipose tissues (27). The results of another study showed that EPA, DHA and docosapentaenoic acid (DPA) and total n-3 PUFA were significantly increased in 2% SALmate- and 5% of salted chickens compared with control and ZnB were obtained (p<0.05) (29). The result of present study similar with the results of Liu that *Lactobacillus johnsonii* BS15 supplementation could increased (p<0.05) C18:3n-3, C20:5n-3, C22:6n-3, total polyunsaturated fatty acid (PUFA), n-3 PUFA and PUFA: saturated fatty acid ratio of chicken meat (30).

### The influence of CFO and *L. casei* WB 315 on HDL, LDL, cholesterol.

The results showed that while the highest LDL level was present in P0, which is different from all the treatments, the lowest LDL levels were found in the treatment of P3. The highest cholesterol level of meat was found at P0 control which is different from all other treatments while the lowest cholesterol meat was produced at P3. These results indicate that the combination of lactic acid isolated bacteria and CFO were capable of lowering the cholesterol level in chicken meat. It’s cholesterol was significantly (p<0.05) lower in *L. casei* WB 315 - supplemented (86.87%) compared to 112.25% in the control chicken. The low cholesterol in broilers which consume crude fish oil compared to the control caused by the feed with fish oil containing high omega-3 fatty acids in the diet impact the concentration of cholesterol. The other research indicated that giving probiotics containing *Lactobacillus* sp. could decrease cholesterol levels in egg’s quail as much as 10.39% compared to the control without probiotics (31).

The result of this study similar with the results of Liu that *Lactobacillus johnsonii* BS15 supplementation could decreased total cholesterol and triglyceride levels (p< 0.05) of chicken meat (30). The study of Ramasamy et al. (2008) showed that the use of *Lactobacillus* culture in laying hens at 24 and 28 weeks of age significantly reduced cholesterol in egg products, whereas the total lipid content and the fatty acid of egg yolk showed similar results between treatments in ages 24, 28 and 32 weeks (32). The results of Abdullah et al. (2006) also showed that the basal diet + 0.1% *Lactobacillus* culture (1 × 10^9^ viable cells per gram) in fed broilers showed that the lower cholesterol contents of the carcass than the control broilers (33). *Lactobacillus* can contribute to increased cholesterol excretion, namely through the mechanism of probiotics to reduce cholesterol through cholesterol synthesis and increased degradation of cholesterol (34). The main pathway for cholesterol excretion is related to the hepatic synthesis of bile acids from cholesterol (35). Certain lactic acid bacteria also have the ability to produce bile salt hydrolase enzyme, which converts bile salts (36), which causes greater excretion of bile salt (37). In the process of re-enterohepatic circulation of biliary acids, the liver will divide more cholesterol into the bile and less into the bloodstream and increased excretion of cholesterol out of the body cause loss of cholesterol from the tissues (38).

Cholesterol-lowering effects of probiotic bacteria can be ascribed to different mechanisms, such as assimilation of cholesterol by probiotic bacteria, binding of cholesterol to the bacterial cell walls and deconjugation of bile salts. Deconjugated bile salts are less water-soluble, so they are less efficiently reabsorbed compared with their conjugated forms. This phenomenon results in the enhanced excretion of free bile acids in feces leading to the increased requirement for cholesterol, which is a precursor for the synthesis of bile salts. Bile salt hydrolase is an enzyme produced by the intestinal microflora that catalyzes the deconjugation of glycine- or taurine-linked bile salts. Thus, a high BSH (bile salt hydrolase) enzyme activity in the intestine could finally contribute to a reduction in the cholesterol level in blood serum. This is due to the work of BSH enzymes which are produced by probiotic bacteria. Bile salt hydrolase enzyme activity has been detected in *Lactobacillus, Bifidobacterium, Enterococcus, Clostridium* and *Bacteroides* spp. Lactobacilli and *Bifidobacteria* are routinely used as probiotic strains, while *Bacteroides, Clostridium* and *Enterococcus* spp. are also commensal inhabitants of the gastrointestinal tract in human and animals (39–41).

## CONCLUSION

The results obtained from the experiments indicated that the addition of 0.5% *Lactobacillus casei* WB 315 (1.2 × 10 ^9^ cfu/ml) and 1% CFO of total broiler basal feed after 35 days could increase feed efficiency, improve feed conversion ratio, decrease feed consumption of broiler, increase EPA, DHA and HDL in broiler meat, reduced the LDL and cholesterol in meat broiler chicken.
